# Do measures of gain asymmetry and catch-up saccades improve video head impulse test agreement with caloric results?

**DOI:** 10.1016/j.cnp.2024.07.001

**Published:** 2024-07-09

**Authors:** I. Zay Melville, Kyla Yamsuan, Helen Wu, Peter R. Thorne, Kei Kobayashi, Rachael L. Taylor

**Affiliations:** a Section of Audiology, School of Population Health, University of Auckland, Auckland, New Zealand; b Department of Physiology and Centre for Brain Research, University of Auckland, Auckland, New Zealand; c Faculty of Science, School of Psychology, University of Auckland, Auckland, New Zealand; d New Zealand Dizziness and Balance Centre, Auckland, New Zealand; e Eisdell Moore Centre for Hearing and Balance Research, University of Auckland, New Zealand

**Keywords:** Vestibular disorders, Video head impulse testing, Caloric testing, Semicircular canal function, Ménière’s disease

## Abstract

**Objective:**

To investigate the relative sensitivity and agreement of caloric testing and video head impulse test (vHIT) across four groups of vestibular disorders.

**Methods:**

Caloric and vHIT results of 118 patients with either Ménière’s disease, vestibular neuritis/labyrinthitis, vestibular migraine, or vestibular schwannoma were retrospectively analyzed. vHIT gain, gain asymmetry, and catch-up-saccades (≥100°/sec) were compared with reference limits of 91 controls.

**Results:**

Abnormal caloric results and vHIT gain were recorded in 57.6 % and 33.1 % of patients, respectively. Consideration of all three measures increased vHIT sensitivity to 43.2 %, and concordance with caloric results improved from 66.1 % to 70.3 %. A significant interaction effect confirmed the relationship between tests depended on the diagnosis (p = 0.013). Vestibular migraine and vestibular neuritis/labyrinthitis produced similar results on both tests, usually normal and abnormal respectively. Vestibular schwannoma produced more caloric abnormalities than vHIT gain but not compared with catch-up-saccades and gain asymmetry; Ménière’s disease produced more caloric abnormalities than all vHIT measures. When vHIT was normal (all measures), a 37 % canal paresis was 90 % specific for Ménière’s disease.

**Conclusions:**

Rates of vHIT catch-up-saccades and gain asymmetry can improve sensitivity and concordance with caloric testing, but this is disease-dependent.

**Significance:**

vHIT outcome measures are complementary to the caloric test and each other.

## Highlights

• Measures of gain asymmetry and catch-up saccades improve caloric and vHIT concordance in vestibular neuritis and vestibular schwannoma.
• Discordant test results were most common in Ménière’s disease, irrespective of which vHIT outcome measure was used.
• When all vHIT measures were normal, a canal paresis >37 % identified Ménière’s disease with 90 % specificity.

## 1 Introduction

The assessment of horizontal semicircular canal (HSC) function is an integral component of the vestibular diagnostic workup. Temperature-regulated water or air irrigated into the external ear canal provides the basis for caloric testing (Bárány, 1906), an example of a non-physiological test of HSC function. More recently, head impulse testing involving small, fast, unpredictable head turns was introduced, providing a physiological stimulus for vestibular hair cell activation (Halmagyi and Curthoys, 1988). Both tests evoke a vestibulo-ocular reflex (VOR) that can be recorded using infrared goggles, providing ear-specific information about HSC function (MacDougall et al., 2009).

While both caloric and video head impulse testing (vHIT) indicate HSC function, they differ in their underlying physiology, methodology, and associated technical pitfalls. Caloric testing indicates low-frequency function (Barin, 2016a). Dependent on temperature conductivity to the inner ear fluids, it is susceptible to individual differences in the anatomy of the outer/middle ear and surrounding temporal bone. This susceptibility makes the strength of the stimulus reaching the vestibular receptors impossible to quantify accurately (Curthoys et al., 2016). In contrast, vHIT involves a measurable high-frequency stimulus (head rotation), affording a degree of control over the magnitude of vestibular stimulation. However, errors can arise if the goggles are incorrectly placed or slip during testing (Curthoys et al., 2016, Halmagyi et al., 2017). The distance of the patient from the fixation target (Judge et al., 2018) and speed of head impulse delivery (McGarvie et al., 2015a) also influence results, and VOR gain, the ratio of eye to head velocity, depends on the recording device and method of calculation (Janky et al., 2017, Pogson et al., 2019). These factors, if unaccounted for, could affect vHIT sensitivity and specificity.

It has been suggested that vHIT may be less sensitive than caloric testing to HCS dysfunction (Bell et al., 2015, Blödow et al., 2014, Burston et al., 2018). However, few studies have explored vHIT measures aside from gain using an appropriately matched control group. When canal function is impaired, the VOR will fail to compensate for head movement, prompting additional saccadic eye movements to “catch-up” and re-establish visual fixation. These so-called catch-up saccades (CS) provide an additional indirect indicator of dysfunction (Du et al., 2021, Jerin et al., 2019, Korsager et al., 2017, Perez-Fernandez and Eza-Nunez, 2015, Yang et al., 2018). However, it is important that CS are distinguished from physiological saccades and other saccadic intrusions which are sometimes recorded in healthy controls.

The interpretation of caloric and vHIT results also requires consideration of the site of lesion and disease pathophysiology. Abnormal caloric results with normal vHIT have been described in several peripheral vestibular disorders, whereas an abnormal vHIT with normal caloric is more prevalent in central disorders (Lee et al., 2020). Dissociated results (normal vHIT and abnormal caloric) are particularly common in Ménière’s disease (MD) and could represent a diagnostic marker of the condition (Blödow et al., 2014, Hannigan et al., 2021, McCaslin et al., 2015). However, the specificity of this pattern to MD is not yet clear.

Here, we investigate the relative sensitivity of vHIT and caloric testing in four groups of vestibular disorders, considering vHIT normal limits for gain, gain asymmetry (GA), and CS. We questioned whether consideration of all three measures might improve vHIT sensitivity and agreement with caloric test results. In addition, we investigated the discriminative accuracy of the caloric/vHIT dissociation in identifying Ménière’s disease.

## 2 Methods

Data were collected and analyzed in accordance with the Code of Ethics of the World Medical Association (2013 Declaration of Helsinki). A retrospective study of existing patient data was approved by the University of Auckland Human Participants Ethics Committee UAHPEC (reference 021388) with a waiver for patient consent. As a prospective cohort, control participants were studied with written informed consent, approved by the UAHPEC (references 021388, 022855 and 017509).

### 2.1 Control group for vHIT

Ninety-one community-dwelling adults (46 female and 45 male; mean age 44 ± 18 years; range 18–79 years) were prospectively recruited as a control group for vHIT. Participants reported normal balance and had no history of vestibular, neurological, or ocular disease. Individuals with severe visual refractive errors (<−5.0 Dioptre) were excluded.

### 2.2 Patients

Horizontal vHIT and caloric test results from a single specialist vestibular clinic, collected on the same day over four years, were retrospectively reviewed. Four groups were studied: Definite Ménière's disease (MD), definite or probable vestibular migraine (VM), vestibular schwannoma (VS), and vestibular neuritis (without hearing loss) or labyrinthitis (with hearing loss) (VN/VL). MD and VM were diagnosed using criteria of the AAOHNS and/or Bárány Society, and tested inter-ictally (Lempert et al., 2012, Lopez-Escamez et al., 2015); gold standard MR imaging identified VS. Patients who had experienced an acute vestibular syndrome lasting >24 h attributed to a peripheral cause comprised the VN/VL group, tested at least three weeks following their vertigo attack (mean = 13.3 months). Excluded from the study were diagnoses of bilateral MD, VN/VL or VS, middle ear pathology, and previous ablative surgery, including gentamicin.

### 2.3 Video head impulse test (vHIT)

Patients and controls were tested using an ICS impulse device (Otometrics, Taastrup, Denmark) while seated 1.0–1.3 m in front of a visual fixation target. Horizontal head impulses of unpredictable timing and direction were performed using the “hands-on-head” method. Traces with significant artifact from suspected blinks or loss of pupil tracking were manually deleted; the study included only cases with at least 15 interpretable left and right head impulses with peak head velocities of 120–300°/s.

Mean gain was calculated within the vHIT software (version 4.1) from the ratio of the area under the curve (AUC) for eye and head velocity. Gain asymmetry (GA) was based on the Mantokoudis formula: %GA = 100 * (1 – (lower gain/higher gain)). To distinguish the side with the weaker response, GA with lower left gain was assigned a negative value, and lower right gain, a positive value.

Rates of CS with peak velocity ≥ 100°/s were determined offline from exported CSV files using custom MATLAB software. This velocity criterion excluded small fixational saccades, which can be difficult to distinguish from CS (Pogson et al., 2019). For each set of head impulses: CS rate = Total CS / Total HI, where total HI represents the number of left- or rightward head turns. Rates >1.0 indicate that some head impulses generated more than one saccade ≥100°/s.

Patient vHIT results were classified as ‘normal’ or ‘abnormal’ based on reference limits determined from control participant data. For MD, VN/VL and VS, ‘ipsilesional’ and ‘contralesional’ distinguished the affected and unaffected side. VM responses were recorded as from the ‘right’ or ‘left’ ear, since the pathophysiology of VM is incompletely understood and could involve either or both ears.

### 2.4 Caloric tests

Patient data included bithermal caloric test results (ICS Chartr 200 VNG AirCal, GN Otometrics), recorded following irrigation of each ear canal with airflow of 8 L/min at 24° and 50° C for 60 s. Patients were tested under the usual standard of clinical care, which included a bedside eye movement examination, recordings of gaze-holding in light and dark, otoscopy and tympanometry. Mental alerting tasks helped maintain arousal, and at least 7 min separated the start of each irrigation. The percentage canal paresis (%CP) was determined from the nystagmus peak slow phase velocity using the Jongkees’ formula (Jongkees et al., 1962). For consistency with vHIT, a negative and positive CP indicated a left and right-sided weakness, respectively. A CP outside the range ±25 % was considered abnormal.

### 2.5 Statistical analysis

Data were analyzed using SPSS software (version 28, IBM Corporation, USA). General Linear Mixed Models (GLMM) or Generalized Estimating Equations (GEE) investigated the effects of head impulse direction and age on gain and CS in controls, while controlling for the effects of head velocity as a covariate. Clinically relevant normal reference limits based on either the mean ± 2SD (normally distributed data) or %range (non-normally distributed data) were then determined for vHIT gain, GA and CS.

Test outcomes for the patient groups were categorized as normal or abnormal and compared using a repeated measures Generalized Estimating Equation (GEE) assuming an exchangeable covariance structure. Independent variables were the disease group and the type of outcome measure (vHIT gain, GA, CS rate, and caloric CP). Significance was determined by a p-value <0.05 after correction for multiple comparisons (sequential Bonferroni method).

Correlations and agreement between vHIT and caloric results were explored using cross-tabulations of categorical data. This information was used to build profiles of results characteristic of each vestibular disorder. Receiver operating characteristic (ROC) analysis determined the usefulness of an abnormal caloric CP in the presence of a normal vHIT in discriminating MD from the other diagnoses.

## 3 Results

### 3.1 vHIT in healthy controls

#### 3.1.1 VOR gain and gain asymmetry

**Fig. 1:**
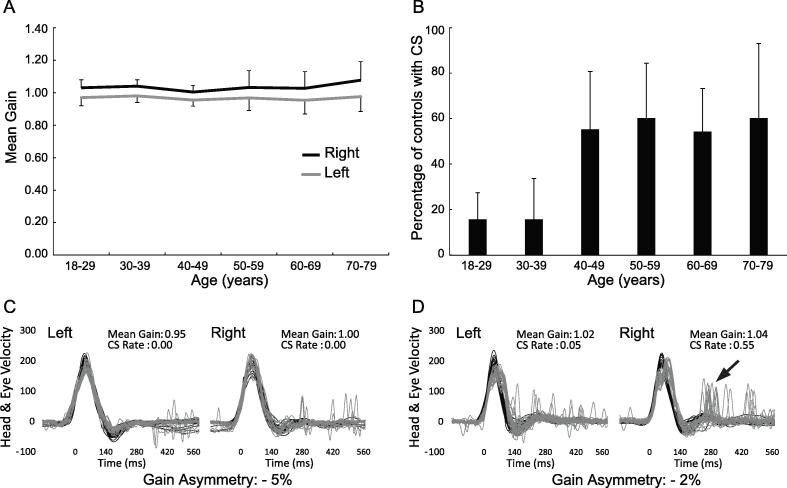
**Video head impulse results in healthy controls.** Gain results (mean ± 2SD) for each decade of age (3rd to 8th) are represented separately in (A) for the left (grey) and right (black) ears. Data for three participants aged 18–19 have been combined with those from the 3rd decade (20–29). The bar graph in (B) shows the percentage of participants from each decade with one or more catch-up saccades (CS) meeting the velocity threshold of 100°/sec. Traces in (C) and (D) contrast the head (black) and inverted eye (grey) velocity recordings of a younger (36-year-old) and older (69-year-old) control. The CS for the younger control in (C) are all below 100°/sec. More prominent CS falling above and below 100°/sec (black arrow) are evident for the older control in (D).

Gain results for the control group are summarised in Fig. 1A. On average, gain for head impulses to the left (0.96 ± 0.06) was significantly lower than to the right (1.03 ± 0.04), giving a mean difference of 0.062 (95 % CI: 0.052–0.072; *F*_1,96.346_ = 154.134; *p* < 0.001). Mean gain was unaffected by age (*F*_5,86.785_ = 0.727; *p* = 0.605), and there was no significant interaction between age and head impulse direction (*F*_5, 84.370_ = 1.313; *p* = 0.267). However, as indicated by the error bars, gain became more variable above age 49-years. Normal limits for gain and GA (Table 1) were therefore calculated separately for age categories 18–49 and 50–79.

**Table 1:** Age-referenced mean (and 95% normal limits) of vHIT Gain and Gain Asymmetry.

	Left Gain	Right Gain	GA (%)
18–49	0.97 (0.88–1.05)	1.03 (0.94–1.12)	−5.80 (−12.39–0.80)
50–79	0.96 (0.80–1.13)	1.04 (0.83–1.25)	−7.14 (−16.80–2.53)

Limits represent the mean ± 2SD. Positive and negative values for gain asymmetry (GA) denote a weaker response (lower gain) for right and left directed head impulses, respectively.

#### 3.1.2 Rates of catch-up saccades

Rates of CS were significantly affected by age (χ^2^_(df=5)_ = 19.252; *p* = 0.002). CS with velocity ≥ 100°/s were rare in participants below age 40, whereas over half the participants above this age had one or more CS meeting this criterion (Fig. 1B, C and D). Post-hoc comparisons against the oldest decade confirmed significant differences for the two youngest groups (*p* = 0.012 and 0.032). Normal limits for CS rates were therefore based on the 97.5th percentile for age categories of 18–39 (Median = 0; upper limit = 0.22) and 40–79 (Median = 0.04; upper limit = 0.55). Since there was no effect of head impulse direction (χ^2^_(df=1)_ = 0.565; *p* = 0.452), or direction by age interaction (χ^2^_(df=5)_ = 6.595; *p* = 0.253), CS data for left and right impulses were combined.

### 3.2 vHIT and caloric results in patients with a vestibular disorder

There were 118 patient records (64 women and 54 men, mean age 53 years, range 21–79 years) fulfilling the study inclusion criteria: 41 with MD (16 right-sided, 18 left-sided); 31 with VM; 38 with VN/VL (20 right-sided, 18 left-sided), and 8 with VS (n = 8; six right-sided, two left-sided). Demographic details, vHIT and caloric test results are summarised in Table 2.

**Table 2:** Summary of demographic and test results of 118 patients in each disease group (n).

	MD (n = 41)	VN/VL (n = 38)	VM (n = 31)	VS (n = 8)
Female: Male	14:27	22:16	24:7	4:4
Mean Age (SD)	51.5 (12.7)	53.4 (14.3)	49.4 (16.2)	65.9 (11.3)
Age Range	25–75	24–79	21–75	45–76
vHIT gain				
Ipsi	0.96 (0.13)	0.66 (0.26)	Left 0.94 (0.12)	0.85 (0.26)
Contra	0.97 (0.12)	0.93 (0.12)	Right 1.01 (0.11)	1.02 (0.15)
vHIT CS				
Ipsi	0.04 (0–0.41)	1.33 (0.33–1.81)	Left 0.00 (0–0.10)	0.94 (0.40–1.41)
Contra	0.03 (0–0.15)	0.05 (0.00–0.25)	Right 0.00 (0–0.04)	0.11 (0.01–0.20)
vHIT %GA	9.0 (5.8)	31.3 (23.2)	7.5 (5.5)	20.3 (17.5)
Caloric %CP	39.2 (22.8)	61.2 (33.7)	17.7 (17.2)	56.9 (31.9)

vHIT Gain, Gain Asymmetry (GA) and Caloric Canal Paresis (CP) are represented as mean (SD); Rates of Catch-up saccades (CS) indicate the median (IQR). Gain asymmetry is represented as an absolute value. Descriptive statistics for gain and CS are shown for ipsilesional (Ipsi) and contralesional (Contra) ears, except for in vestibular migraine where they are provided for left and right ears. MD, Ménière’s disease; VN/VL, vestibular neuritis/labyrinthitis; VM, vestibular migraine; VS, vestibular schwannoma.

**Fig. 2:**
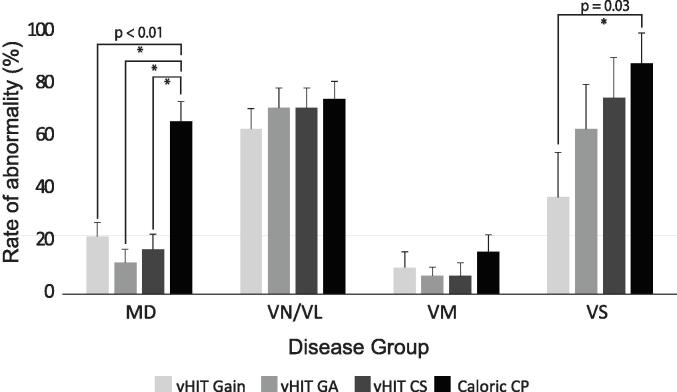
**Rates of abnormal vHIT and caloric CP results across disease groups.** Bars indicate the percentage of abnormalities + SEM of abnormalities for each outcome measure (GA = Gain asymmetry; CS = Catch-up saccades; CP = Canal paresis). For unilateral vestibular disorders (Ménière's disease, vestibular neuritis/labyrinthitis and vestibular schwannoma), gain and CS represent ipsilesional abnormalities. For vestibular migraine, both left and right ear results are included.

Abnormal vHIT (gain only) was recorded in 33.1 % of the entire group. This increased to 43.2 % when GA and CS were included. The rate of abnormal caloric CP was 57.6 %. There were significant main effects of disease group (χ^2^_(df=3)_ = 39.701, p < 0.001) and the type of outcome measure (χ^2^_(df=3)_ = 18.080, p < 0.001). Abnormalities were overall highest for VN/VL and lowest for VM, and caloric testing produced more abnormalities overall than the three vHIT measures. However, a significant group by outcome measure interaction (χ^2^_(df=9)_ = 20.962, p = 0.013) confirmed the relationship between results depended on the diagnosis (Fig. 2). Caloric testing in MD produced significantly more abnormalities than all three vHIT measures (*p* < 0.001). In contrast, there were no significant differences for VM and VN/VL and only weak evidence of a difference for VS between vHIT gain and caloric CP (*p* = 0.030).

### 3.3 Relationship between caloric and vHIT outcome measures

Categorized vHIT and caloric test outcomes (normal or abnormal) for the entire group were all significantly correlated (p < 0.001). Correlations were strongest among the vHIT measures (rho range: 0.655 to 0.757); vHIT and caloric test outcomes were moderately correlated (rho range: 0.409 to 0.494). Agreement between caloric and vHIT was 66.1 % when based on gain, increasing to 70.3 % on considering all measures. However, this depended on the diagnosis, being strongest in VN/VL and weakest in MD (Table 3), yielding different results profiles for each disorder (Table 4).

**Table 3:** Percentage agreement between caloric test outcome and vHIT results for each vestibular disorder.

	Gain	Signed GA	CS	vHIT (any)
MD	41 % (17)	34.1 % (14)	41 % (17)	41 % (17)
VM	81 % (25)	84 % (26)	84 % (26)	77 % (24)
VN/VL	87 % (33)	89 % (34)	89 % (34)	95 % (36)
VS	37.5 % (3)	62.5 % (5)	75 % (6)	88 % (6)
Total	66.1 % (78)	66.9 % (79)	70.3 % (83)	70.3 % (83)

Values represent the % concordance (and total number) for test outcomes coded as both normal or both abnormal for vHIT compared with caloric testing. The first three columns are based on the result for each individual vHIT outcome measure; the last column considers a vHIT abnormality on any one of the outcome measures. Results described in section 3.4 for two patients were conflicting (lateralizing to opposite ears) and classified as discordant, despite both tests demonstrating abnormalities.

**Table 4:** Profiles of vestibular test results.

	Normal CP Normal vHIT	Normal CP Abnormal vHIT	Abnormal CP Normal vHIT	Abnormal CP Abnormal vHIT
**A) Based on Caloric CP and abnormal vHIT (Gain only)**				
MD	26.8 (11)	7.3 (3)	**51.2 (21)**	14.6 (6)
VN	23.7 (9)	0.0 (0)	13.2 (5)	**63.2 (24)**
VM	**77.4 (24)**	6.5 (2)	12.9 (4)	3.2 (1)
VS	12.5 (1)	12.5 (1) ƚ	**50.0 (4)**	25.0 (2)

	Normal CP Normal vHIT	Normal CP Abnormal vHIT	Abnormal CP Normal vHIT	Abnormal CP Abnormal vHIT
**B) Based on Caloric CP and abnormal vHIT (Gain OR GA OR CS)**				
MD	24.4 (10)	9.8 (4)	48.7 (20)*	17.1 (7)
VN	21.1 (8)	2.6 (1)	2.6 (1)	**73.7 (28)**
VM	**74.2 (23)**	9.7 (3)	12.9 (4)	3.2 (1)
VS	12.5 (1)	12.5 (1) ƚ	12.5 (1)	**62.5 (5)**

Values for each diagnosis represent the percentage (and number) of patients demonstrating each pattern of results, based only on vHIT gain (A), then on considering the results of all three vHIT measures (B). Bold indicates the most common profile for each group. *Includes one MD patient where the vHIT GA incorrectly lateralised to the unaffected ear. ƚ Includes one VS patient whose caloric CP implicated the unaffected ear.

### 3.4 Accuracy of asymmetry measures and contralesional abnormalities in MD, VN/VL, and VS

Of 66 patients with a unilateral vestibular diagnosis and abnormal CP and/or GA, the abnormality lateralised to the affected ear in all but two cases. One case with MD in the right ear had a −16 % GA (left-ear weakness); there were no other contralesional vHIT abnormalities and a 38 % CP lateralised to the affected side. Another case with a left VS had a 33 % right CP; all three vHIT measures were abnormal only for the left ear.

Nine VN/VL patients had abnormal contralesional gains (n = 7) and/or CS (n = 5) in addition to ipsilesional abnormalities; however, gain was always lower (mean = 0.42 vs 0.76) and CS rate higher (mean = 1.50 vs 0.84) on the lesioned side. Gain was reduced bilaterally in two MD patients, and two MD patients had CS or reduced gain only for the contralesional ear. Further review of their clinical data confirmed absence of bilateral vestibulopathy on caloric testing, and all but one had preserved contralesional vestibular evoked myogenic potentials.

### 3.5 Profiles of results for different vestibular disorders

**Fig. 3:**
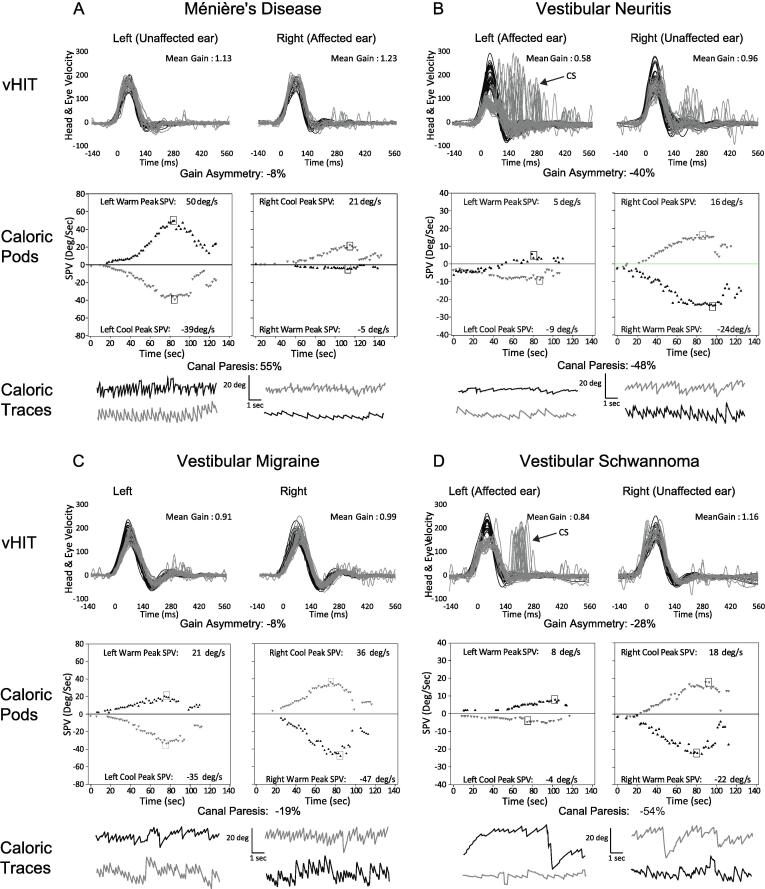
**Patterns of caloric and vHIT results in different vestibular disorders.** For each of A-D, vHIT head (black) and inverted eye (grey) velocity traces are contrasted with results of caloric testing, presented as a plot of the slow-phase velocity (SPV) of nystagmus over time, and nystagmus eye movement recordings for warm (black) and cool (grey) irrigations. Results in (A) are from a patient with Ménière’s disease affecting the right ear showing a normal vHIT with a 55% right canal paresis. In (B), abnormal caloric and abnormal vHIT gain, GA and CS are shown for a patient with a left vestibular neuritis. Significant CS exceeding normal limits are indicated for the affected ear (arrow). Additional, lower velocity CS falling within the range of healthy controls are evident for head impulses toward the contralesional side. Normal caloric and vHIT results are shown for a patient with probable VM in (C). The vHIT and caloric results in (D) are from a patient with a left VS. The vHIT gain is normal, whereas CS, GA and caloric results are abnormal for the lesioned side.

Profiles of test results in Table 4 are for comparisons between caloric CP and vHIT gain (A), then on consideration of gain, GA, and CS (B). When comparing caloric CP with vHIT gain, most MD and VS patients showed an abnormal CP with normal vHIT; for VM and VN/VL, both tests were respectively normal and abnormal. Inclusion of GA and CS decreased test discordance from 33.9 % (n = 40) to 29.7 % (n = 35). Profiles for VN/VL, MD and VM were unchanged, although proportions differed. For VS, the dominant profile of *normal* vHIT (gain) with *abnormal* caloric changed to an *abnormal* vHIT and *abnormal* caloric. Examples of results seen in each disorder are shown in Fig. 3(A–D).

### 3.6 Specificity of the caloric (abnormal)/vHIT (normal) dissociation for Ménière’s disease

**Fig. 4:**
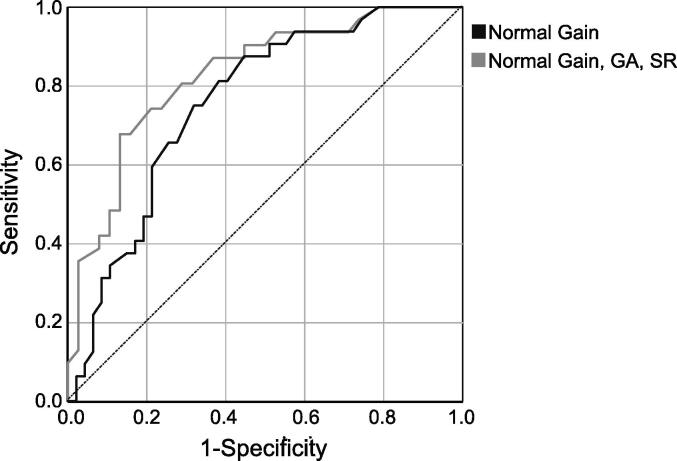
Receiver operating characteristic (ROC) curves comparing the discriminative accuracy of caloric canal paresis in the presence of normal vHIT gain (black; n = 79), and normal vHIT on all outcome measures (grey, n = 68).

The ROC analyses in Fig. 4 for 79 patients with normal vHIT gain and 68 patients with normal vHIT gain, GA, and CS, indicate fair (AUC = 0.759; 95 % CI: 0.653–0.864) and good (AUC = 0.821; 95 % CI: 0.721–0.921) discrimination, respectively. For patients with normal gain, a caloric CP >25 % was 72 % specific and 66 % sensitive for MD. A higher CP threshold of 50 % improved specificity to 90 % while decreasing sensitivity to 31 %. For patients with normal vHIT on all measures, CP >25 % was 84 % specific and 67 % sensitive; a CP threshold of 37 % achieved superior specificity (90 %), and sensitivity of 47 %.

## 4 Discussion

Since vHIT was introduced, gain has been used as a direct measure of horizontal VOR function and is the basis of most vHIT and caloric test comparisons. This study compared results of caloric testing with vHIT using three quantitative measures (gain, catch-up saccades (CS) and gain asymmetry (GA)) across four groups of vestibular disorders. vHIT results were classified according to ear-specific, age-referenced normative data to determine whether inclusion of rates of CS and GA might make vHIT more sensitive to dysfunction, improving the agreement between tests. The data suggest that these additional vHIT measures are complementary and can in some cases improve sensitivity and concordance with caloric testing, but that this is disease specific.

### 4.1 vHIT in healthy controls and derivation of normal reference limits

Accurate classification of patient results requires an understanding of vHIT characteristics in a comparable healthy population tested under similar conditions. Our results confirm previous studies using the ICS device (Bell et al., 2015, Janky et al., 2017, Matino-Soler et al., 2014, McGarvie et al., 2015a, Rambold, 2015) demonstrating lower gain for head impulses to the left. This directional bias has been attributed to a combination of geometrical differences, owing to the camera position over the right eye, and anatomical differences in the neural pathways underlying adducting versus abducting eye movements (McGarvie et al., 2015a, Weber et al., 2008). Consistent with other studies (Matino-Soler et al., 2014, McGarvie et al., 2015a), age did not significantly affect average gain. However, increased variability was seen in older decades, with some individuals aged over 49-years demonstrating slightly lower or higher gain than their younger counterparts. Lower gains may be a sign of pre-symptomatic presbyvestibulopathy. Skin laxity contributing to goggle slip has been suggested as a potential reason for higher gain in older adults (Li et al., 2014), while calibration errors and incorrect head/goggle orientation are other sources of gain variability that should be considered (Curthoys et al., 2023, Mantokoudis et al., 2015). Taken together, our results support the use of ear-specific, age-referenced gain and gain asymmetry limits.

Compared with gain, CS in healthy controls have been less studied. It is not uncommon for controls to demonstrate small corrective saccades, and the frequency, velocity and amplitude of these saccades increase with age (Anson et al., 2016, Janky et al., 2018, Pogson et al., 2019). Our criteria considered both CS velocity and frequency, confirming that the prevalence of CS ≥100°/sec increases from about the 5th decade. It is difficult to know if this is due to increased fixational instability, which can occur in compensatory and anti-compensatory directions, or small eye position errors due to subtle age-related changes in the VOR. Irrespective of the origin, these results suggest that age should be considered when interpreting CS in the patient population.

### 4.2 Comparison of caloric and vHIT results in the patients with vestibular disorders

The overall yield of the vHIT outcome measures was similar, and agreement with caloric results (normal or abnormal) improved only slightly from 66.1 % to 70.3 % on considering all measurements. Our discordance rate (29.7 %) is higher than in other studies using a gain limit of 0.8. Lee et al. (2020), in a group of heterogeneous disorders, found discordant results in 18.1 %, while Hannigan et al. (2021) using a more stringent CP of 30 %, found discordance in only 5.6 %, mainly in patients with MD. Given the recognized relationship between MD and the pattern of abnormal CP/normal vHIT, different rates of test agreement can be explained, at least in part, by differences in study populations. Samples like ours, with a high proportion of MD patients (35 % in our study), can be expected to have higher discordance rates. For this reason, further discussion of results will be provided separately for disorders showing good test agreement and those with more frequent discrepancies.

#### 4.2.1 Diseases demonstrating good test agreement (VN/VL and VM)

Good test agreement was found in patients with VN/VL and VM. In VN/VL, an abnormal caloric CP was typically associated with low vHIT gain, high GA and CS rates, with optimal sensitivity on considering all three measures. VN preferentially affects the superior vestibular nerve, causing abnormalities on both tests during the acute phase of the syndrome (Bartolomeo et al., 2014). In one study, vHIT gain normalized more rapidly than caloric CP, producing fewer abnormalities when both tests were repeated after one month (Bartolomeo et al., 2014). Our results indicate good test agreement even in the chronic phase, which might be due to the use of our own normative data, including GA and CS. Supporting this suggestion, Yang et al. reported increased rates of abnormal vHIT in chronic VN on considering GA and CS, although caloric results were not provided for comparison (Yang et al., 2018).

Interestingly, VN/VL was the disorder most often associated with additional contralesional vHIT abnormalities. These deficits were mild, and other vestibular function tests showed no evidence of bilateral pathology. Contralesional gain abnormalities have been described previously in VN, mainly in patients with severe ipsilesional abnormalities (Blödow et al., 2013). In such instances, they are probably due to loss of the inhibitory response from the lesioned canal/nerve, thereby removing the disinhibitory influence on contralesional vestibular nucleus neurons for head turns toward the intact side. Thus, vHIT can detect small inaccuracies in the contralesional VOR, which could have functional relevance for the patient, but which are not detected on caloric testing. Attention to other vestibular test results remains important for excluding evolving bilateral pathology.

VM also showed good agreement between vHIT (all measures) and caloric results with low rates of abnormalities on both tests. Three other studies yielded similar low rates (<30 %) (Blödow et al., 2014, Hannigan et al., 2021, Rambold, 2014), although higher rates of caloric abnormalities have been reported (Mahringer and Rambold, 2014). There is evidence of central vestibular dysfunction in VM (Na et al., 2019, von Brevern et al., 2005), therefore, it is conceivable that VM might produce fewer abnormalities in tests of peripheral vestibular function. However, the effects of VM on vestibular function are not fully understood. Labyrinthine ischemia due to vasospasm of the internal auditory artery has been proposed, and investigators have raised the possibility of a pathophysiologic link with MD (Radtke et al., 2002), which could include endolymphatic hydrops (Oh et al., 2021). These and other mechanisms could account for the small percentage of VM patients with abnormal vHIT or caloric results.

#### 4.2.2 Ménière’s disease

The frequent recording of an abnormal caloric with normal vHIT in MD is consistent with other studies using vHIT gain. We further demonstrate that this pattern prevails despite the inclusion of GA and CS. The dissociation is therefore unlikely to be due to a sensitivity issue affecting vHIT gain and supports the view that it is due to the underlying pathophysiology.

McCaslin et al. (2015) suggested the dissociation could reflect selective loss of type II vestibular hair cells, as demonstrated in one temporal bone study of MD (Tsuji et al., 2000). These cells are innervated by a greater number of afferents with a regular rate of spontaneous discharge, suitable for encoding sustained low velocity stimulation associated with caloric testing. An alternative explanation posits that the abnormal caloric CP is due to dilation of the HSC membranous duct, reducing the hydrostatic drive across the cupula (McGarvie, et al., 2015b). Cupula responses to natural stimuli, i.e., head acceleration, are unimpeded; therefore, fewer vHIT abnormalities are expected. This theory is supported by computer simulations (Rey-Martinez et al., 2017) but is contradicted by a recent temporal bone study which found no evidence of duct dilation in MD patients (Shen et al., 2022). Instead, an abnormal caloric result was associated with herniation of the otolith membrane into the HSC (Shen et al., 2022). The investigators proposed a selective effect on low frequency HSC function, although vHIT results were not reported to confirm the effects on high frequency function.

#### 4.2.3 Vestibular schwannoma

Similar to MD, VS patients demonstrated lower rates of abnormal gain compared to caloric CP. While this finding requires cautious interpretation due to the small sample, it is consistent with at least two studies showing rates of abnormal caloric CP up to 2-fold higher than for vHIT gain (Brown et al., 2019, Tranter-Entwistle et al., 2016). It is possible the slow growth of the VS, and gradual loss of hair cells and afferents, facilitates more effective central vestibular compensation to high velocity head movements. Studies exploring VOR rehabilitation protocols suggest gain adaptation is optimal when small degrees of retinal image slip are introduced incrementally (Schubert et al., 2008), as would occur with a slow growing VS. Daily head movements could, for small lesions, be enough to restore gain to within the range of controls. Some VS patients develop endolymphatic hydrops (Hizli et al., 2016), which could preferentially affect low frequency HSC function. However, contrasting with MD, we found the test dissociation usually resolved on considering CS and GA.

Few studies have directly compared vHIT and caloric results of patients with VS with those of MD. Hannigan et al. (2021) identified two of six patients with VS who showed a dissociation similar to their MD patients. Other studies focusing only on VS demonstrate dissociated results in 30–42 % of cases (Blödow et al., 2015, Tranter-Entwistle et al., 2016). One of these studies included GA and, like us, confirmed an increase in vHIT sensitivity (Blödow et al., 2015). These and our results highlight the value of considering vHIT measures other than gain. Future studies would benefit from more detailed analysis of vHIT outcome measures in VS, contrasting the findings with other vestibular disorders.

### 4.3 Dissociated caloric and vHIT as a diagnostic marker for MD

Our results support the suggestion that the pattern of an abnormal CP with normal vHIT could be a marker for MD (Hannigan et al., 2021, McCaslin et al., 2015). However, as discordant results were sometimes seen in other disorders, we asked the question; if a patient has a normal vHIT on all outcome measures, how large does the CP need to be to indicate MD with reasonable confidence? Our results suggest a slightly higher CP (37 %) than is used clinically was necessary to achieve good specificity of 90 %. Sensitivity was not high at 48 %, partly because MD patients can have normal results on both tests. Future studies which include a broader range of vestibular diagnoses, including those with endolymphatic hydrops, will better inform the specificity of this pattern in the broader patient population. Repeat testing in patients with recurrent episodic vertigo of less certain diagnosis is also required to confirm the value of the caloric/vHIT dissociation in the early MD stages.

### 4.4 Other clinical considerations

Differences in vHIT and caloric results can occur due to the underlying pathophysiology, as in MD. However, they can also be due to technical error, and neither test is immune to producing false-positive or −negative results. Technical difficulties inherent in caloric testing are well documented (Barin, 2016a). Likewise, goggle slip and other artifacts, which could mask a gain reduction, are a primary consideration for vHIT (Curthoys et al., 2016, Halmagyi et al., 2017, Mantokoudis et al., 2015). Analysis of CS, an indirect measure of VOR function, could in some instances be informative since they are less likely to be affected by slip of the recording goggles. However, CS are also influenced by individual subject factors (Curthoys et al., 2023). Measures of GA, an inter-aural comparator, may be less prone to subject factors and as our data demonstrate, an abnormal GA can discriminate between affected and unaffected ears with high accuracy. When all caloric and vHIT measures agree, confidence in the presence/absence of a lesion increases. When disagreement occurs, clinicians should consider the potential reasons for this, whether a technical error, subject factor, or a consequence of disease pathophysiology.

### 4.5 Study limitations

A limitation of the vHIT results of controls is that data were collected by four different testers. However, all were trained by the same clinician with over 10 years vHIT experience, and the objective was to simulate the clinical setting, which often involves different testers. Secondly, although mean head velocity was accounted for in the statistical analysis, separate normative data for different head velocities were not provided, which could improve diagnostic accuracy (McGarvie et al., 2015a). Thirdly, we do not distinguish between early and late CS, and CS amplitudes, timing, and dispersion were not explored. More studies are needed which compare these CS properties in healthy controls and across multiple diseases to confirm the CS metrics which are most useful clinically.

For caloric testing, the absence of a control group is a potential limitation, although the CP of 25 % used in this study is considered an acceptable limit of normality (Barin, 2016b). Calculations of directional preponderance and total nystagmus slow phase velocity would likely have contributed to the diagnostic profiling of the vestibular disorders. However, the significance of a large directional preponderance without a CP is unclear (Halmagyi et al., 2000), and total slow phase velocity can be influenced by central mechanisms and subject factors (Jacob, 1988, Torok, 1970). As we were interested in ear-specific hypofunction, we studied only CP.

Additional limitations arise from the retrospective analysis of patient results, which will have been influenced by clinic referral patterns and demographic factors. Disease groups were not equally represented, and the small sample of VS prevented analysis of any relationship with tumour size. Patients were also at different stages in their disorder or recovery, which can affect the relationship between results (Altunay and Ozkarakas, 2020, Mahringer and Rambold, 2014). Importantly, our conclusions relate only to the vestibular disorders that were studied.

## 5 Conclusions

The relative sensitivity, and relationship, between vHIT and caloric testing is disease dependent. Consideration of GA and CS produced small improvements in test agreement, mainly in patients with VS or VN/VL. Patients with VM show low rates of abnormalities, whereas those with MD often have abnormal caloric results with normal vHIT, irrespective of the outcome measure. When vHIT was normal on all measures, a CP > 37 % was 90 % specific for MD. Measures of vHIT gain, GA and CS are complementary to the caloric test and each other.

## Conflict of interest statement

RT is employed for contracted clinical services by the New Zealand Dizziness and Balance Centre (NZDBC), from which patient data were reviewed.

## CRediT authorship contribution statement

**I. Zay Melville:** Data curation, Investigation, Writing original, Writing review & editing, Formal analysis. **Kyla Yamsuan:** Data curation, Writing review & editing. **Helen Wu:** Data curation, Writing review & editing. **KK:** Software, Writing review & editing. **Peter R. Thorne:** Writing review & editing, Supervision. **Kei Kobayashi:** Conceptualization, Methodology, Data curation, Writing review & editing, Formal analysis, Supervision.
